# Tumor-induced lymph node alterations detected by MRI lymphography using gadolinium nanoparticles

**DOI:** 10.1038/srep15641

**Published:** 2015-10-26

**Authors:** S. C. Partridge, B. F. Kurland, C.-L. Liu, R. J. Y. Ho, A. Ruddell

**Affiliations:** 1 Seattle Cancer Care Alliance, Seattle WA USA; 2 Department of Radiology, University of Washington, Seattle WA USA; 3 Department of Biostatistics, University of Pittsburgh, Pittsburgh, PA; 4 Department of Pharmaceutics, University of Washington, Seattle WA USA; 5 Department of Comparative Medicine, Seattle WA USA; 6 Fred Hutchinson Cancer Research Center, Seattle WA USA

## Abstract

Contrast-enhanced MRI lymphography shows potential to identify alterations in lymph drainage through lymph nodes (LNs) in cancer and other diseases. MRI studies have typically used low molecular weight gadolinium contrast agents, however larger gadolinium-loaded nanoparticles possess characteristics that could improve the specificity and sensitivity of lymphography. The performance of three gadolinium contrast agents with different sizes and properties was compared by 3T MRI after subcutaneous injection. Mice bearing B16-F10 melanoma footpad tumors were imaged to assess tumor-induced alterations in lymph drainage through tumor-draining popliteal and inguinal LNs versus contralateral uninvolved drainage. Gadolinium lipid nanoparticles were able to identify tumor-induced alterations in contrast agent drainage into the popliteal LN, while lower molecular weight or albumin-binding gadolinium agents were less effective. All of the contrast agents distributed in foci around the cortex and medulla of tumor-draining popliteal LNs, while they were restricted to the cortex of non-draining LNs. Surprisingly, second-tier tumor-draining inguinal LNs exhibited reduced uptake, indicating that tumors can also divert LN drainage. These characteristics of tumor-induced lymph drainage could be useful for diagnosis of LN pathology in cancer and other diseases. The preferential uptake of nanoparticle contrasts into tumor-draining LNs could also allow selective targeting of therapies to tumor-draining LNs.

Gadolinium contrast-enhanced MRI lymphography is being developed for analysis of lymphatic vessel drainage function in a variety of disorders including cancer^1^, lymphedema^2^, and rheumatoid arthritis^3^. For oncology, MRI lymphography is of particular interest for image-guided mapping of sentinel lymph nodes (SLNs) draining tumors, and for assessment of SLN hypertrophy^4^,^5^. Imaging after interstitial injection of gadolinium contrast media has been used to identify draining LNs in rabbits^6^, dogs^7^, and mice^8^. In several types of human cancers, MRI lymphography using conventional contrast media such as Gd-DTPA also shows potential to detect SLNs^9^,^10^,^11^.

Tumors can induce alterations in lymph drainage that could be exploited to non-invasively guide diagnosis and treatment. First, the tumor-draining LN (TDLN) often exhibits hypertrophy^4^, which indicates immune cell accumulation^12^,^13^. Another early TDLN alteration is the extensive growth of TDLN lymphatic sinuses^14^,^15^,^16^, which is associated with strongly increased lymph drainage through the TDLN^12^,^17^. For example, murine footpad melanoma-draining LNs exhibit increased lymphatic sinuses and lymph flow through the draining popliteal LN by optical imaging after subcutaneous injection of quantum dots or fluorescent nanoparticles^12^, and by MRI after injection of dimeglumine gadopentate (Gd-DTPA) contrast agent^17^. Pre-neoplastic lymphomas also exhibit LN lymphatic sinus growth and increased lymph flow by optical imaging^16^. This lymphangiogenesis and increased lymph flow may be a characteristic of TDLNs with metastatic potential, as mice bearing benign tumors do not develop significant LN lymphatic sinus growth^18^. In humans, pathology studies suggest that TDLN lymphangiogenesis predicts poor prognosis in breast^19^, oral squamous carcinomas^20^,^21^, and rectal cancers^22^. Increased or altered lymph drainage also shows promise to identify human melanomas^23^ or skin cancers^24^ with poor prognosis. Metastases overgrowing the TDLN can also block drainage through that LN region^6^,^25^. Thus MRI lymphography has potential not only to accurately identify the TDLN, but also to provide information on tumor metastatic potential.

One challenge with the use of low molecular weight gadolinium contrasts for lymphography or angiography is their rapid diffusion out of the vessels, limiting the time and resolution of imaging. However, the lymphatic vasculature uniquely is able to take up nanoparticles into blind-ended initial lymphatic vessels, for specific labeling of the lymphatic vasculature^26^, and also for retention of contrast media to allow longer imaging with increased resolution. Larger gadolinium-containing nanoparticles such as those composed of dendrimers have also shown promise in rodent angiography and lymphography studies^8^,^27^. Another approach used gadolinium-coated lipid nanoparticles (Gd-LNP), which showed improved performance in MRI angiography in rats and monkeys^28^. This formulation could potentially be translated for application to humans due to its biocompatible design^29^. Gd-LNP is primarily excreted via the biliary route rather than via the kidneys^28^, which could minimize potential gadolinium nephrotoxicity^30^. Gd-LNP holds particular promise for subcutaneous MRI lymphography, as the average particle diameter is roughly 75 nm, so that the contrast could be selectively taken up into and then retained within the lymphatic vasculature^26^.

Another gadolinium contrast agent that shows potential to improve vessel imaging is gadolinium fosveset trisodium (Gd-FVT), which forms a small nanoparticle of ~4 nm diameter by binding to albumin after injection^31^, to extend imaging time by MRI angiography^32^. We recently demonstrated the utility of Gd-FVT for 3T MRI lymphography, using the B16-F10 footpad melanoma model. Gd-FVT uptake labeled the enlarged tumor-draining popliteal LN as well as the contralateral uninvolved popliteal LN, although the tumor-induced increase in flow was not captured using this high resolution scanning protocol^33^. The pattern of contrast agent uptake was altered in TDLNs, as Gd-FVT labeled the cortical and medullary margins of TDLNs, while in uninvolved LNs contrast was restricted to the cortex^33^. These findings suggested that MRI lymphography could be developed to identify tumor-induced alterations in lymph drainage.

In this study, we compared the performance of 3 types of gadolinium contrast media in tumor-bearing mice, to identify the optimal size and/or type of agent for MRI lymphography of normal or tumor-draining LNs and lymphatic vessels. The B16-F10 footpad melanoma model was used for these studies as it reliably increases lymph flow through the popliteal tumor-draining LN (TDLN), with the contralateral uninvolved popliteal LN serving as an internal control^12^. First, low molecular weight Gd-DTPA contrast was tested, as this has been previously used for MRI lymphography^7^,^34^. We previously found that Gd-DTPA was not able to resolve LN anatomy at 1.5T^17^, however we hypothesized that resolution and relaxation differences of MRI at 3T could improve detection of LNs with this contrast agent. Second, the larger lipid nanoparticle Gd-LNP was tested as it shows improved angiography performance by several criteria^28^. Third, Gd-FVT was tested as an example of a small nanoparticle which previously allowed visualization of LNs^33^. Each contrast agent was assessed quantitatively to characterize the pattern of distribution of each contrast agent through LNs and lymphatic vessels. Quantitative analysis compared tumor-draining and contralateral uninvolved LNs for differences in lymph flow, measured by change in integrated density following contrast injection.

## Results

### Distribution pattern of contrast media uptake into the popliteal lymph nodes

**Figure 1:**
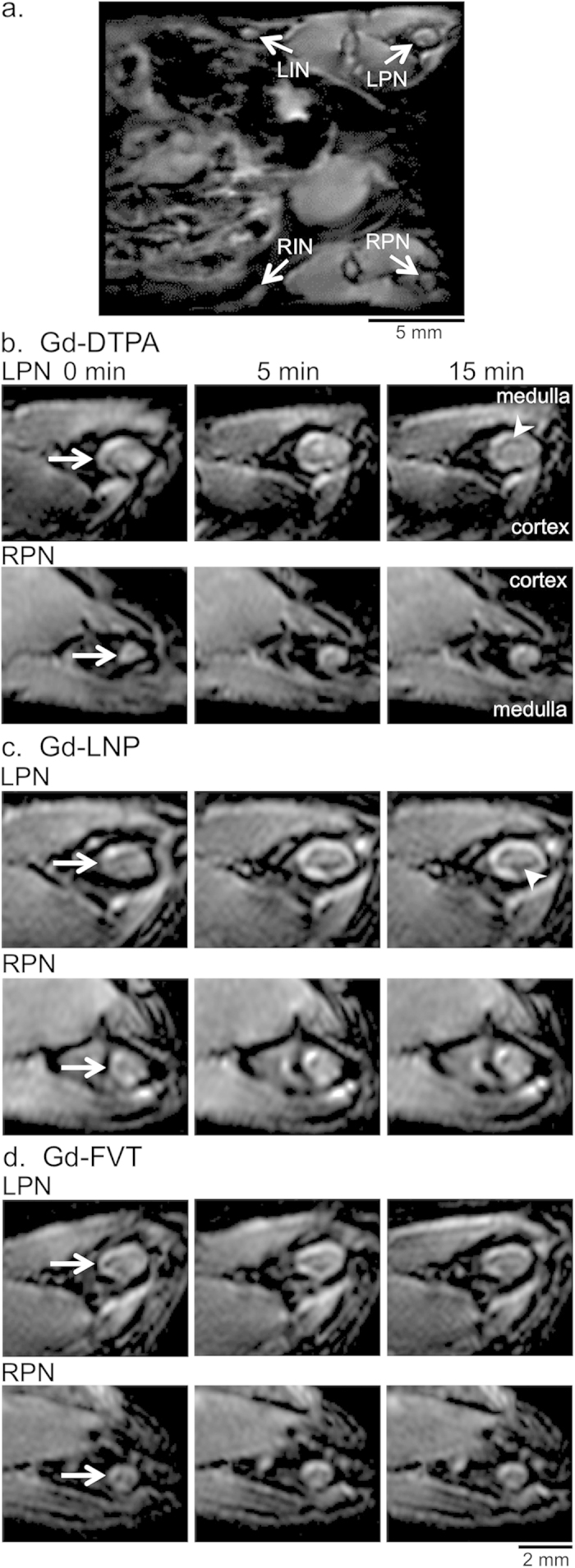
Gd-LNP contrast media detects increased lymph drainage through the tumor-draining popliteal lymph node. (**a**) Full field-of-view oblique MIP image, illustrating the *in vivo* locations of the left and right popliteal (LPN and RPN, respectively) and inguinal LNs (LIN and RIN) that were analyzed in the study. The MIP was generated from 5 min post- GD-LNP images, in the same animal shown in part c. (**b**) Representative single slice images of LPN and RPN (arrows) from pre-contrast (0 min) and from 5 and 15 minute post-contrast scans after Gd-DTPA injection demonstrate modest LN enhancement after contrast injection. The orientation of the cortex and medulla is indicated. Arrowhead indicates the dark artifacts arising after contrast agent injection. (**c**) Single slice images of popliteal LNs after Gd-LNP injection show higher uptake into the LPN at 5 and 15 min. (**d**) Single slice images of popliteal LNs after Gd-FVT injection show uptake into the LPN and RPN at 5 min after contrast agent injection. Scale bars are indicated.

The 3T MRI lymphography performance of Gd-DTPA was compared with that of Gd-LNP and Gd-FVT in mice bearing B16-F10 tumors in the left rear footpad. Contrast agents injected into the feet drain to the left and right popliteal LNs (LPN, RPN), and then to the left and right inguinal LNs (LIN, RIN, Fig. 1a). Hypertrophy of the tumor-draining left popliteal LN (LPN) relative to the uninvolved right popliteal LN (RPN) could be appreciated in pre-contrast images, however subcutaneous injection of Gd-DTPA slightly improved delineation of the LPN margins (Fig. 1b). Gd-DTPA uptake into the LN margins was detectable at 5 min and at 15 min after injection. Gd-LNP nanoparticles (71–75 nm diameter) were readily taken up into the tumor-draining LPN, strongly enhancing the LN margins at 5 and at 15 min after injection, while the RPN showed less enhancement (Fig. 1c). We previously reported uptake of the intermediate size Gd-FVT contrast agent into the popliteal LNs using the same B16-F10 foot tumor model and 3T scanner protocols^33^. A representative example shows intermediate uptake of contrast into the LPN margins, and also into the RPN (Fig. 1d). The tumor-draining LPN as consistently enlarged in all 18 of the mice studied (median volume 2.6 mm^3^, range 1.2 to 5.0 mm^3^), was 3.3 times larger on average than the RPN (median 0.8 mm^3^, range (0.4 to 1.4 mm3; p < 0.001 by Wilcoxon signed rank test), in agreement with our previous studies^12^.

Dark artifacts also appeared in varying locations within the LPNs after contrast injection, and these were a feature of the TDLN in 5 of 6 mice injected with Gd-DTPA (e.g. arrowhead, Fig. 1b), in 5 of 6 mice injected with Gd-LNP (arrowhead Fig. 1c), and in 2 of 6 mice injected with Gd-FVT^33^. However, these dark regions were not observed in any of the non-draining RPN post-contrast images, suggesting that they are associated with contrast agent accumulation in the tumor-draining LN.

**Figure 2:**
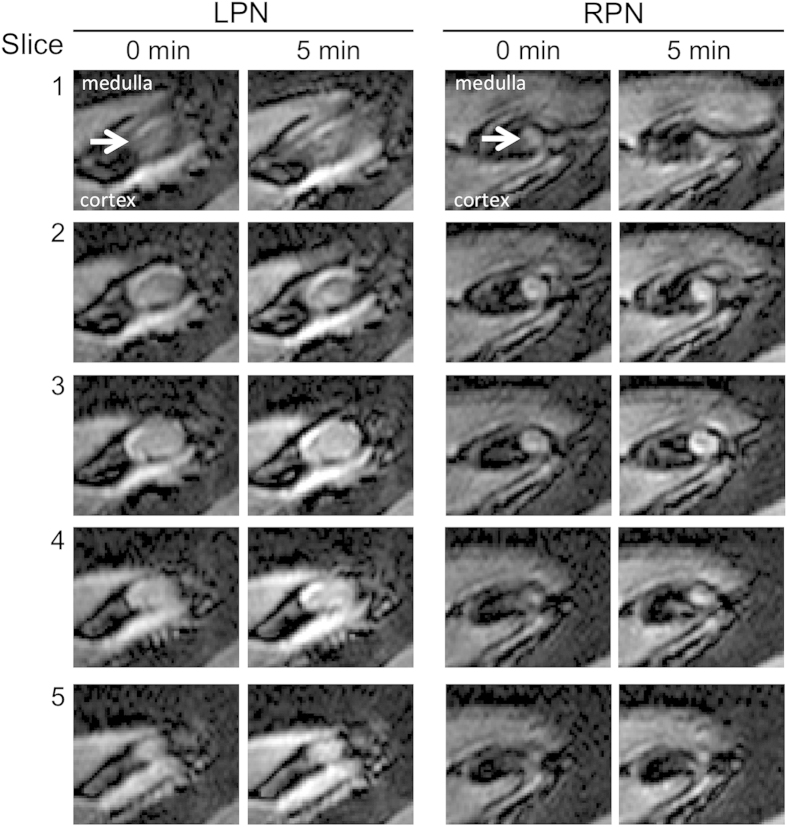
Slice-by-slice illustration of Gd-LNP uptake distribution through lymph nodes. Serial images sampled through the entire left and right popliteal LNs before and 5 min after subcutaneous injection of Gd-LNP. Comparison of pre- and post-contrast images show Gd-LNP enhancement in the medulla and cortex of the LPN, while contrast uptake is more restricted to the cortex of the RPN of all slices. Arrows indicate the popliteal LNs. Note that the RPN images have been flipped vertically to facilitate comparison of the LPN and RPN anatomy.

The pattern of uptake of both contrast agents was distinct in the tumor-draining LPN and non-draining RPNs of the mice. In the popliteal LN of young mice, the cortex roughly extends over the medial LN, while the medulla extends over the lateral half of the LN^17^. All three contrast agents were mainly distributed on the cortical margin of the control RPN at 5 and at 15 min after contrast injection (Fig. 1). However, the LPN consistently acquired contrast around the margins of the medulla as well as the cortex, a TDLN characteristic that was more readily detected with the stronger enhancement provided by Gd-LNP contrast media. These distinct patterns of contrast agent uptake were identified throughout the LNs, as illustrated by sampling images through the entire LNs from a different Gd-LNP-injected mouse example (Fig. 2).

### Quantitative assessment of contrast media uptake into the tumor-draining popliteal LN

The uptake of Gd-DTPA and Gd-LNP into the popliteal LNs was quantified by measurement of the integrated density (sum of pixel values in the LN)^17^, which takes into account both the change in signal intensity and the volume over which it occurs. This enabled comparison of total contrast uptake into the normal size RPN versus the enlarged tumor-draining LPN^17^. While Gd-DTPA (Supplementary Fig. 1a), Gd-LNP (Supplementary Fig. 1b), and Gd-FVT contrast uptake^33^ was higher in the LPN than in the RPN, these differences were not statistically significant using whole LN assessments. We observed that the dark artifacts arising in post-contrast TDLNs dampened post-contrast integrated density measures, and therefore reduced our ability to detect the tumor-induced contrast agent uptake. To minimize artifact effects, we instead focused on assessment of the “hot-spot” intense regions of contrast uptake in the LN margins, where the majority of contrast uptake occurs (Fig. 1). The 90^th^ percentile of pre-contrast values was selected empirically as a threshold for hot-spot analysis, as it gave the greatest separation between the LPN and RPN (based on post-contrast changes in integrated density) with all three contrast media tested (Supplementary Fig. 2).

**Figure 3:**
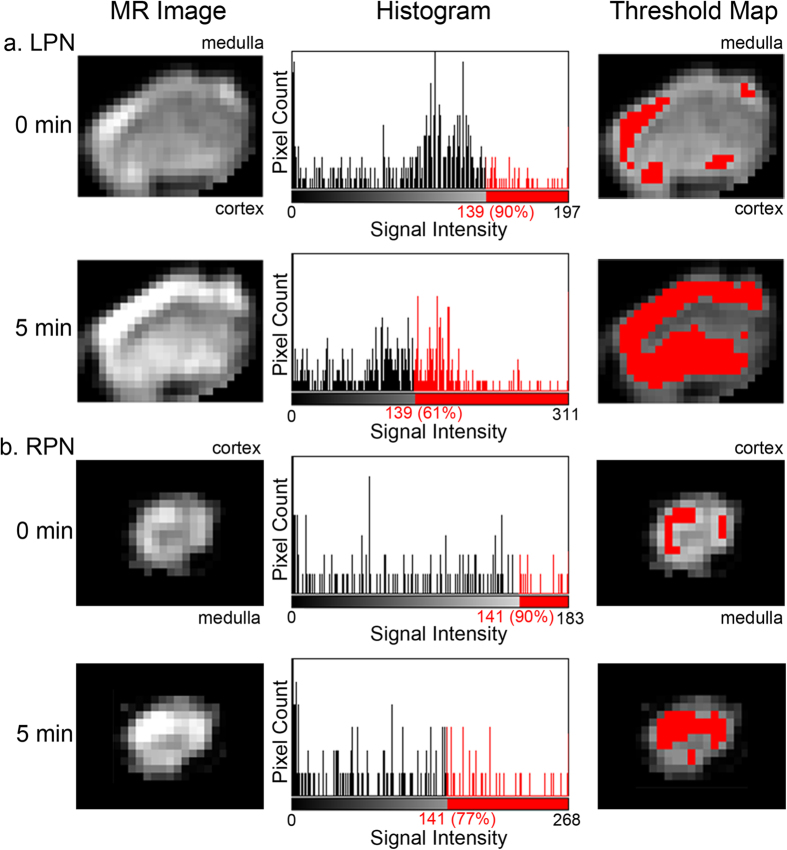
Hot-spot analysis of contrast agent uptake into the popliteal lymph nodes. For each LN, histogram analysis was used to determine the 90^th^ percentile pre-contrast threshold, and to select only post-contrast voxels above the same threshold value for integrated density calculations and analysis. Shown are representative MR images before (0 min) and after (5 min) Gd-LNP injection (left panels), associated LN signal intensity histograms (center panels), and thresholded maps (right panels). (**a**) In the LPN, the 90^th^ percentile pre-contrast value of 139 was determined from the histogram, which corresponded to the 61^st^ percentile of 5 min post-contrast pixel values. Pixels with values above 139 and included in this analysis are shown in red on threshold maps. (**b**) The process was repeated for the RPN, where the 90^th^ percentile pre-contrast value was 141, which corresponded to the 77^th^ percentile of 5 min post-contrast pixel values.

The number of pixels meeting the 90^th^ percentile integrated density threshold in the LPN were increased at 5 min after Gd-LNP injection, as illustrated in the red-highlighted threshold map of Fig. 3a. In contrast, the RPN showed less contrast enhancement, with fewer pixels above the 90^th^ percentile threshold in post-contrast images (Fig. 3b, threshold map). Furthermore, the proportion of pixels above the 90^th^ percentile highlighted in red in the histograms (Fig. 3b) was smaller for the RPN (mean 12%) than for the LPN (mean 26%; p < 0.001 Wilcoxon signed rank test), confirming that these bright pixels were less abundant in the RPN. Pixels with signal intensity above the 90% threshold, corresponding to enhancing regions, outlined the margins of the medulla and cortex of the LPN at 5 min after contrast injection (Fig. 3a, threshold map), while the RPN showed maximal contrast uptake in the cortex (Fig. 3b, threshold map).

The integrated density followed a similar trajectory over time for all 3 contrast agents, for both the LPN and RPN: average integrated density was lowest pre-contrast, was greater at 5 min post-contrast, and was similar at 15 min post-contrast. Pre-contrast integrated density ranged from 48,000–691,000 with a mean of 217,000. Post-contrast integrated density was lowest for the low-molecular-weight agent Gd-DTPA, with mean integrated density 540,000 (range 217,000–1,855,000), and higher for Gd-LPN (mean 860,000, range 33,000–2,418,000) and Gd-FVT (mean 836,000, range 136,000–2,396,000).

**Figure 4:**
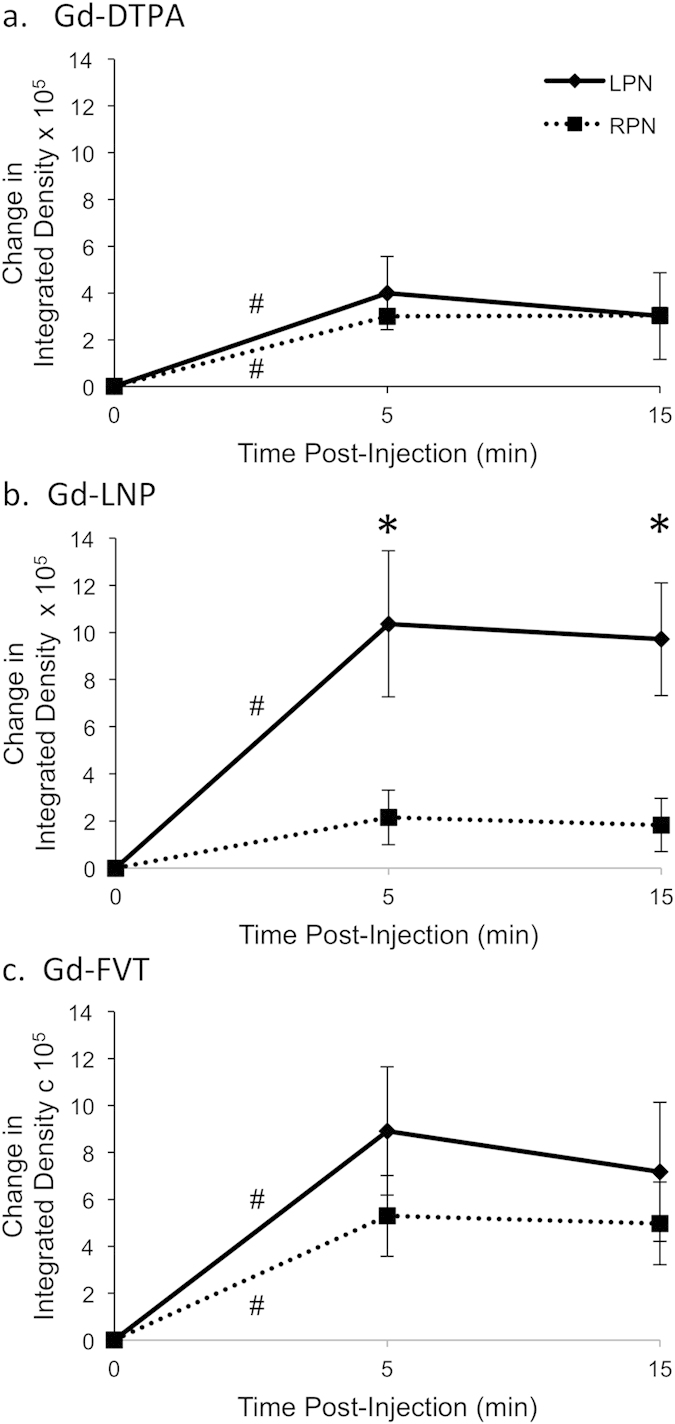
Quantitation of contrast agent uptake in tumor-draining and uninvolved popliteal lymph nodes. Integrated density values over each popliteal LN were calculated using the 90^th^ percentile threshold method. Change from pre-contrast integrated density is summarized for groups of 6 mice receiving each contrast agent, with standard error bars displayed at each timepoint. (**a**) The integrated density of Gd-DTPA contrast uptake into ROIs increases within 5 min after injection in the LPN (^#^p = 0.01) and RPN (^#^p = 0.006), as estimated in a linear mixed effects regression model. (**b**) The integrated density of Gd-LNP contrast uptake increases within 5 min after injection for the tumor-draining LPN (^#^p = 0.009), but not for the RPN (p = 0.24). Moreover, the contrast uptake into the LPN is significantly higher than into the RPN at both 5 and 15 min after injection (*p < 0.01). (**c**) Gd-FVT uptake into the LPN and RPN 5 min after injection is statistically significant (^#^p = 0.02 for both), although the difference in contrast uptake between the LPN and RPN is not significantly different at 5 min (p = 0.14) or 15 min (p = 0.52).

Uptake of the contrast agent caused an increase in integrated density within 5 minutes for both LPN and RPN (Fig. 4), with the exception of the RPN for Gd-LNP (average increase 215,000, p = 0.24). Increase in LPN integrated density ranged from an average increase of 399,000 for Gd-DTPA (p = 0.01) to an average increase of 1,036,000 for Gd-LNP (p = 0.009).

The primary analysis examined whether contrast uptake was greater in the LPN (involved node) than for the RPN (uninvolved node). Change in Gd-DTPA integrated density between pre-contrast and 5 min post-contrast was similar for the LPN and RPN (Fig. 4a, Tukey-Kramer adjusted p = 0.85). Gd-LNP showed a strong increase in contrast uptake in the LPN relative to the RPN at 5 min (Fig. 4b, p = 0.006). From a linear mixed effects model with random mouse effect and LPN versus RPN change in integrated density estimated for each timepoint, the estimated increase in integrated density from pre-contrast to 5 min was 821,000 greater for the LPN than for the RPN (95% confidence interval 227,000–1,416,000). While average change in Gd-FVT integrated density was higher in the LPN than in the RPN (Fig. 4c, average difference of 362,000), this difference was not statistically significant (p = 0.14, 95% confidence interval 91,000 lower – 814,000 higher difference for LPN than RPN).

The t = 15 min post-contrast timepoint was also compared, where the rate of contrast uptake slowed and the lymph nodes showed reduced levels of contrast agent relative to 5 min, presumably due to lymphatic drainage to more central LNs. Again, only Gd-LNP showed significantly greater contrast agent uptake in the tumor-draining LPN relative to the RPN (Fig. 4b, Tukey-Kramer adjusted p = 0.008), while Gd-DTPA (Fig. 4a, p = 1.00) and Gd-FVT (Fig. 4c, p = 0.52) exhibited similar contrast agent uptake in LPN vs. RPN. Thus Gd-LNP was the only contrast agent able to significantly demonstrate tumor-induced contrast agent uptake at t = 5 min and the continued higher accumulation of contrast agent within the LN at t = 15 min.

### Quantitative assessment of contrast media uptake into the second-tier inguinal lymph nodes

**Figure 5:**
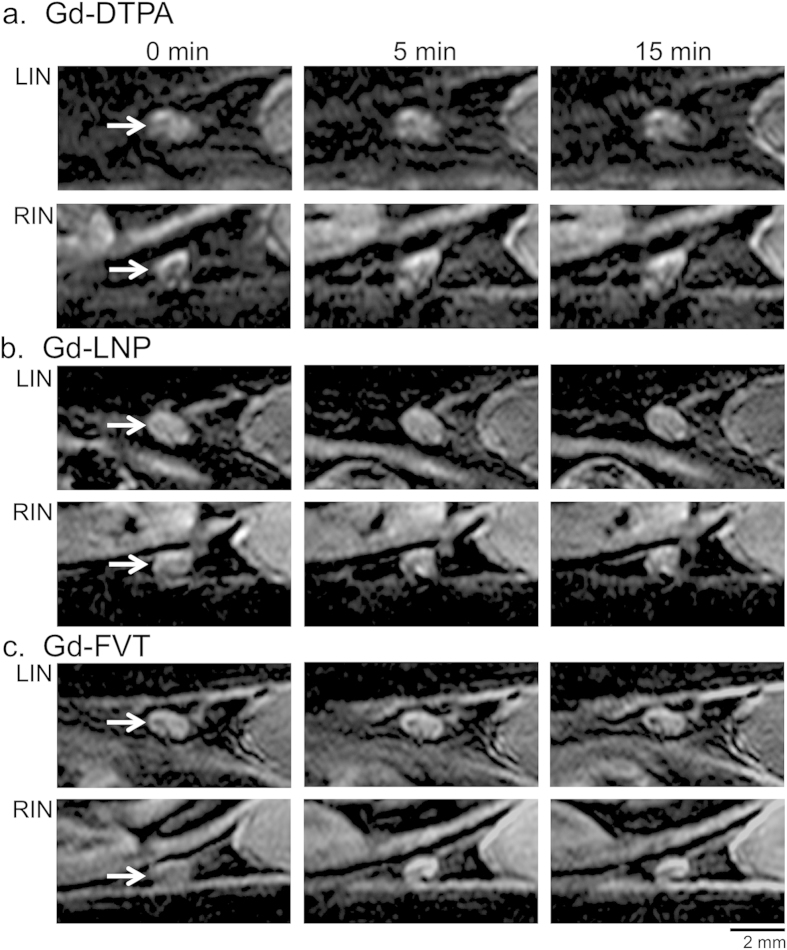
Contrast agent uptake in the inguinal lymph nodes. (**a**) The uptake of Gd-DTPA into the LIN and RIN (arrows) is shown in single slice examples. Gd-DTPA uptake is minimally detected in the RIN, and not in the LIN. (**b**) Gd-LNP is taken up into the RIN but not into the LIN. (**c**) Gd-FVT contrast agent accumulates in the RIN and LIN, at 5 and at 15 min after injection. Scale bars are indicated.

**Figure 6:**
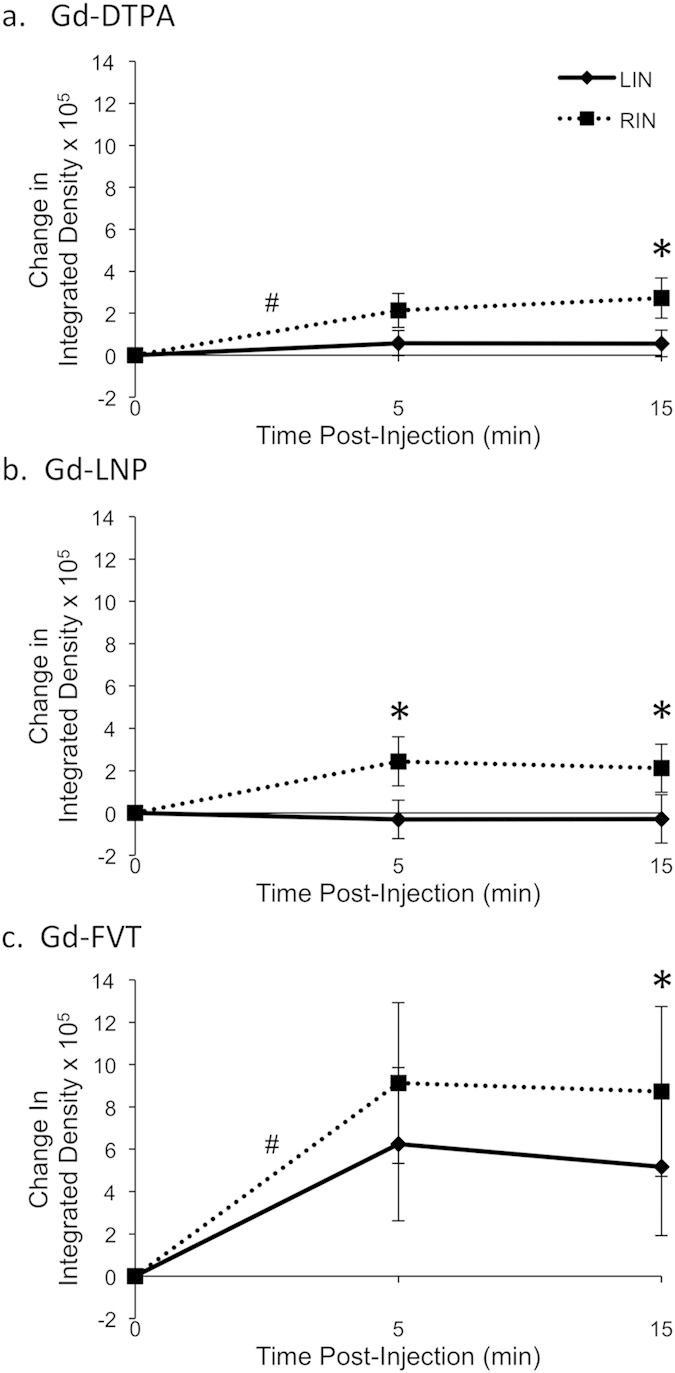
Quantitation of contrast agent uptake in inguinal lymph nodes. Integrated density values in each inguinal LN were calculated using the 90^th^ percentile threshold method. Change from pre-contrast integrated density is summarized for groups of 6 mice receiving each contrast agent, with standard error bars displayed at each timepoint. (**a**) Gd-DPTA uptake into the RIN appears to be greater than into the LIN at 5 min after injection (p = 0.07) and at 15 min after injection (*p = 0.009). (**b**) Gd-LNP also accumulates in the RIN more than the LIN at 5 min after injection (*p = 0.01) and at 15 min after injection (*p = 0.03). (**c**) Similar trends were seen for Gd-FVT, with contrast uptake greater into the RIN than into the LIN at 5 min after injection (p = 0.11) and at 15 min (*p = 0.04). In most cases the integrated density of contrast uptake was not found to increase within 5 min after injection (p > 0.05 in linear mixed effects model), with the exception of the RIN for Gd-DTPA (average 213,000 increase, ^#^p = 0.002) and RIN for Gd-FVT (average 913,000 increase, ^#^p = 0.02).

Lymph from the popliteal LNs drains to the inguinal LNs, and also to the central iliac LNs^17^. Contrast agent uptake into the left (LIN) and right (RIN) inguinal LNs was examined, to test whether tumors also increase lymph drainage into these second-tier LNs. The inguinal LNs were of similar size in all of the mice (Fig. 5), showing no increase in volume of the tumor-draining LIN (median volume 2.5 mm^3^, range 0.6 to 4.6 mm^3^) relative to the RIN (median 2.5 mm^3^, range 0.6 to 3.7 mm^3^; p = 0.55 by Wilcoxon signed rank test). Surprisingly, contrast agent uptake was found to be greater in the RIN than in the tumor-draining LIN (Fig. 5). Quantitation of un-thresholded integrated density values over the whole LN (Supplementary Fig. 3) determined that contrast agent uptake was significantly higher in the RIN versus LIN for Gd-DTPA at 15 min (Tukey-Kramer adjusted p = 0.015) and Gd-LNP at both 5 min and 15 min (p = 0.004 and p = 0.005, respectively). Hot-spot analysis of the brightest 90^th^ percentile pixels further determined that contrast agent uptake into the RIN was significantly higher than into the LIN, for all three contrast agents (Fig. 6). Gd-DTPA contrast agent uptake appeared to be greater in the RIN than in the tumor-draining LIN at 5 min (average 155,000 greater increase in integrated density for RIN versus LIN, Tukey-Kramer adjusted p = 0.07) and 15 min (average 217,000 greater, p = 0.009) after contrast injection (Fig. 6a); Gd-LNP also showed greater post-contrast integrated density increase in the RIN compared to LIN at both 5 min (average 274,000, p = 0.01) and at 15 min (average 240,000, p = 0.03, Fig. 6b). For the intermediate size Gd-FVT, the increase in integrated density was not greater for RIN versus LIN at 5 min (average 289,000, p = 0.11), but was at 15 min (average 357,000, p = 0.04, Fig. 6c).

**Figure 7:**
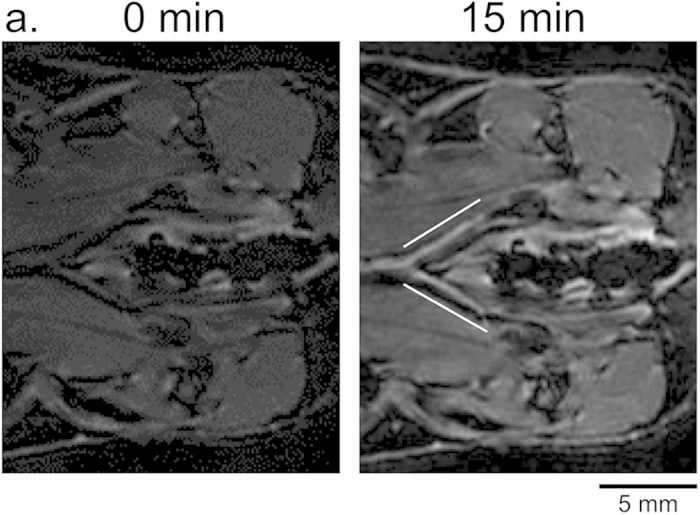
Gd-FVT labels the central lymphatic drainage. Pre-contrast (t = 0 min) MIP of slices in the spine region, and at 15 min after Gd-FVT injection identify the femoral lymphatic vessels (solid lines) draining toward the central iliac LNs. Scale bars are indicated.

**Figure 8:**
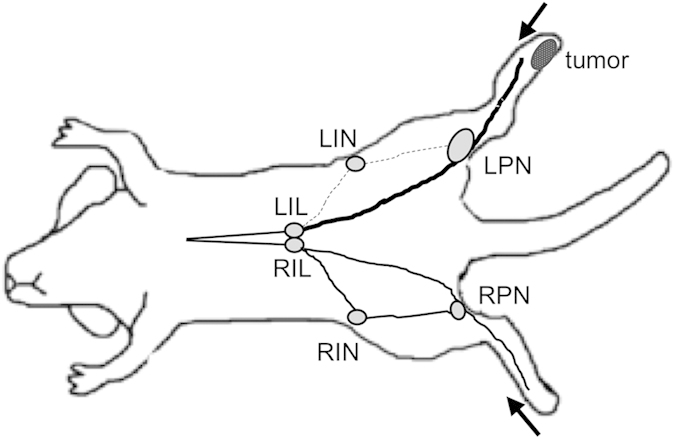
Tumors alter lymph drainage. The lymph drainage pattern in the tumor-bearing left leg is altered relative to the uninvolved right leg. Drainage from the tumor to the LPN increases, continuing on to the left iliac LN (LIL), while the drainage to the LIN is reduced. In the uninvolved right leg, lymph drains normally to the RPN and on to the right iliac LN (RIL) and RIN.

While the left and right inguinal LNs showed differential contrast agent uptake, the femoral lymphatic vessels draining centrally from the popliteal LNs toward the iliac LNs on the midline from the left and right legs demonstrated similar Gd-FVT labeling (Fig. 7, lines). Gd-DPTA also detected both femoral lymphatic vessels^17^, while Gd-LNP was not as useful to detect this drainage. These findings indicate that lymph drainage from the popliteal LNs to the femoral lymphatic vessels is not grossly changed by tumor growth, while drainage to the second-tier tumor-draining LIN is selectively reduced, as illustrated in Fig. 8. Taken together, these findings indicate that tumors not only enhance but also can redirect lymph drainage at a distance from the primary tumor.

## Discussion

MRI lymphography shows promise to identify and map tumor-draining sentinel LNs in animals and in humans. Our comparison of the performance of distinct types of gadolinium contrast agents identified distinct differences in their utility for detection of tumor-draining or uninvolved LNs, summarized in Table 1. Small molecule Gd-DTPA weakly labeled the LNs and was unable to distinguish increased TDLN contrast uptake with the 10 min image acquisition times used in this study, likely because this low molecular weight contrast transits through both LNs within the first minutes after injection^17^. Gd-FVT more effectively labeled the LPN and RPN, although this agent also was not able to significantly distinguish tumor-induced contrast uptake. However, the larger nanoparticle Gd-LNP contrast media behaved very differently in normal and tumor-draining LNs, with the tumor-draining popliteal LN exhibiting strong uptake relative to the uninvolved LN. These comparisons suggest that larger nanoparticle contrasts may be most useful to identify tumor-induced lymph drainage in mice. Due to their large size (~75 nm) and retention in the lymphatic system these nanoparticles can be imaged for longer times to increase resolution for at least 15 min in our studies. Gd-LNP offers several unique advantages, including greatly increased T1 relaxivity^28^ so that a much lower dose may be adequate to label the lymphatic drainage. In fact, in this study the Gd-LNP effectively labeled the TDLN at a gadolinium dose 2.5 times lower that that used for Gd-DTPA or Gd-FVT. Gd-LNP are also primarily eliminated via the biliary route rather than from the kidney^28^, which could reduce the potential for nephrotoxicity^30^. Finally, Gd-LNP can be loaded with more than one kind of agent for theranostic applications combining drug delivery and imaging^35^,^36^. One drawback of Gd-LNP or other large nanoparticle contrast agents could be their inefficient uptake into the second-tier inguinal LNs, which could limit their utility for mapping lymph drainage beyond the sentinel LN (Table 1). In this regard, GD-FVT may be more useful to map drainage through both first- and second-tier LNs.

Our finding that Gd-LNP strongly enhances the tumor-draining LPN while it is not significantly detected in the uninvolved RPN was unexpected, and suggests that tumors not only can increase lymph flow, but also that they somehow activate preferential uptake of nanoparticles into the TDLN. The basis for this selective uptake remains to be determined. The abnormal growth and dilation of the lymphatic vasculature surrounding tumors^37^,^38^ could be required for efficient uptake of these nanoparticles into the lymph drainage. The expanded lymphatic sinuses characteristic of TDLNs^12^,^39^,^40^ could also potentially alter their barrier function to permit accumulation of nanoparticles within the popliteal LN. We previously identified a 20–30 fold increased uptake of nanoparticles of 30 and 52 nm diameter into the tumor-draining popliteal LN in this same B16-F10 model by optical imaging, with delayed and weak uptake into uninvolved contralateral popliteal LNs^12^, providing further support for our finding that larger nanoparticles selectively label TDLNs after subcutaneous injection. This preferential nanoparticle contrast uptake could potentially make a useful diagnostic feature for identification of the first tumor-draining sentinel LN. In addition, this feature could help to target drugs^29^,^35^ or vaccines^41^ to the first TDLN via subcutaneous injection.

The pattern of contrast agent uptake was characteristically altered in the tumor-draining popliteal LN relative to the uninvolved popliteal LN. In the tumor-draining LPN contrast enhancement was detected on the margins of the medulla and cortex, while in the uninvolved RPN contrast uptake was restricted to the cortex at 5 or 15 min after contrast injection. Our previous studies found that B16-10 footpad tumors reliably induce extensive growth of TDLN medullary and cortical lymphatic sinuses, which show a 9-fold increase in area in relative to the non-draining LN^33^. Moreover, fluorescent dye injection into the dorsal toes of these mice and immunostaining of the popliteal LNs for lymphatic sinuses demonstrated that the dye specifically labels the LN lymphatic sinuses for at least 20 minutes after injection^33^. Our finding that the mice injected with all 3 contrast agents exhibited these distinct spatial patterns of lymph drainage demonstrate that this shift in drainage is a consistent feature of TDLNs, which could serve as a surrogate marker of the TDLN lymphangiogenesis induced by these footpad melanomas^12^. Finally, dark artifacts within the tumor-draining popliteal LN were often identified in different LN regions after injection of all of the contrast agents. The cause of these artifacts is not known, although similar dark regions have been identified by Gd-FVT contrast-enhanced MRI angiography of lymph nodes in human rectal cancer^9^,^42^. These dark regions were not caused by melanotic melanoma metastasis in the LPN, as these mice did not contain significant metastases, and the dark artifacts appeared only after contrast injection.

Unexpectedly, the second-tier inguinal LNs which are downstream of the tumor-draining popliteal LNs exhibited reduced rather than increased lymph drainage relative to the uninvolved inguinal LN, with all of the contrast agents (Table 1). Notably, even though Gd-LNP was extensively taken up into the tumor-draining LPN, contrast did not reach the LIN at all. The mechanism for this inhibition remains to be determined. Animal studies have shown that large tumors or metastases can divert or block lymph drainage^43^,^44^, however in this model the small foot tumors are located at some distance from the inguinal LNs. Moreover, none of the LNs contained gross metastases at this early stage, so that metastasis does not explain this shunting effect. Instead, the tumor may somehow influence the efferent lymphatic vessels exiting the tumor-draining popliteal LN to shunt lymph drainage to the central iliac LNs, and reduce drainage to the inguinal LN. The popliteal LN also drains to a small gluteal (or ischiatic) LN^45^, which then continues into the iliac LNs. This LN was not included in the scanning window in most of the mice imaged in this study. We previously demonstrated increased Gd-DTPA contrast agent uptake into this second-tier tumor-draining LN using 1.5T MRI, although it was mis-identified as the inguinal LN in that low-resolution study^17^. Additional high-resolution imaging will be required to determine the mechanism by which tumor-draining inguinal LN drainage is diminished, while flow is increased to the more central gluteal and iliac LNs. Tumor-induced reductions in the pattern of lymph flow arising even before LN metastasis could potentially complicate detection of some of the second-tier LNs draining cancers.

This study suggests several directions for refinement of contrast-enhanced MRI lymphography analysis of lymph drainage. Gd-LNP shows potential to selectively label the first tumor-draining LN. It will be interesting to determine how this selectivity is obtained, and the nanoparticle size range ideal for targeting the sentinel LN. Further analysis could optimize gadolinium agent dose, scanning protocols, and kinetics to measure tumor-induced alterations in lymph drainage, and tumor-responsive changes in the pattern of lymph circulation through LNs. The ability to more finely map and quantify contrast enhancement would be facilitated by the development of a catheter system to remotely deliver contrast agent, so that pre-and post-contrast images can be precisely registered, and image subtraction can be more precisely performed. The 90% percentile threshold for calculation of the brightest hot-spot integrated density was chosen to yield the best separation of contrast behavior in these data, but should be validated in an independent cohort. Finally, it remains to be determined whether Gd-LNP or other nanoparticle contrast agents are preferentially taken up into TDLNs in humans, and if they are safe for subcutaneous injection^10^,^11^.

Our MRI lymphography studies identified several features of lymph drainage consistently altered by tumor growth, detected using this high resolution scanning approach at 3 Tesla. First, tumors were associated with increased uptake of Gd-LNP contrast media into the first tumor-draining popliteal LN, which could be quantified by measurement of thresholded integrated density in popliteal LNs from Gd-LNP-injected mice. Second, the pattern of contrast agent uptake was altered in the tumor-draining popliteal LN. In normal popliteal LNs, the lymph drainage is relatively restricted to the cortical margin, while in the TDLN the expanded lymphatic sinuses deliver contrast agents into the cortex and also the medulla. Third, lymph drainage to the second-tier inguinal LN was paradoxically reduced by tumor growth, suggesting shunting of lymph drainage to more central LNs. These characteristics could be useful not only to identify tumor lymphatic drainage, but may also be useful to assess metastatic potential, as the extent of TDLN lymphangiogenesis appears to predict metastatic potential in murine^18^ and human cancers^19^,^21^,^22^. Further investigation will determine the collective utility of these phenotypes to provide diagnostic criteria for evaluation of tumor-draining LNs in humans.

## Materials and Methods

### Mouse Tumor Model

C57Bl/6 mice (Jackson Laboratories, Bar Harbor, ME, USA) were maintained under specific pathogen-free conditions in microisolator rooms at Fred Hutchinson Cancer Research Center animal facility. Five week-old mice were injected in the left hindleg footpad with 200,000 B16-F10 cells (American Type Culture Collection, Manassas, VA, USA), and in the right hindfoot with saline, as previously described^12^. Six mice were imaged with each contrast agent after 21 to 23 days, when tumors were 2 to 5 mm in diameter. Tumor metastasis was assessed by microscopic inspection of LNs dissected after necropsy, to identify black-pigmented metastases^46^. Experiments were carried out in accordance with approved guidelines, and were approved by the Fred Hutchinson Cancer Research Center and University of Washington Animal Care and Use Committees.

### Preparation and sizing of gadolinium-lipid nanoparticles

Gd-LNP were prepared as described previously^28^. Briefly, DSPC:DMPE-DEPA:DSPE-mPEG2000 (9:1:1 mole ratio) were dissolved in chloroform, and solvent was removed by rotation under a stream of N_2_ gas followed by dessication *in vacuo*. The lipid film was rehydrated in phosphate-buffered saline at 60 °C and particle size was reduced by bath sonication or by extrusion through a 50 nm polycarbonic filter. The resulting nanoparticles were mixed at 55 °C with Gd^3+^ (1:1 DMPE-DTPA:Gd mole ratio) for 20 min, raised back to 60 °C, cooled to room temperature, and stored at 4 °C. Particle diameter was 71–75 nm, determined by photon correlation spectroscopy using a Malvern Zetasizer 5000 (Malvern Instruments, Malvern, United Kingdom).

### Magnetic Resonance Image Acquisition

Mice were imaged on a 3.0-T Philips Achieva clinical MRI scanner (Philips Healthcare, Best, The Netherlands) using a dedicated single-channel solenoid mouse RF coil (Philips Research Laboratories, Hamburg, Germany), as previously described^33^. Animals were anesthetized with 3% isoflurane through an MR-compatible mobile inhalation system (DRE Inc, Louisville, KY) and sedation was maintained during imaging with 2.5% isoflurane. Animals were placed supine on a custom platform in the RF coil, with legs loosely taped to a water-filled 15 ml test tube at the same level to maintain positioning and to optimize magnetic field homogeneity. A landmark was placed to indicate the platform position within the RF coil.

Following localizer scans and pre-contrast T1-weighted scanning, the animal platform was partially slid out of the coil for contrast injection. The dorsal toes of both rear feet were injected subcutaneously with 25 μl of Gd-DTPA (Magnevist; 0.025 mmol/kg; Bayer Pharmaceuticals, Wayne, NJ, USA), Gd-FVT (0.025 mmol/kg; Ablavar: Lantheus Medical Imaging; N. Billerica, MA, USA) or Gd-LNP (0.01 mmol/kg), in sterile HBSS (Invitrogen, Grand Island, NY, USA). The platform was reinserted into the RF coil, aligning the landmark to ensure the platform was returned to the correct position, and post-contrast scans were performed. Mice were euthanized after imaging by 5% isoflurane overdose for 5 min, followed by cervical dislocation and by necropsy. Six mice were imaged and analyzed using each contrast agent.

The MR imaging protocol was optimized to provide high spatial resolution with adequate signal-to-noise and scan time^33^. Imaging was performed using a coronal T1-weighted 3D fast gradient echo sequence with fat suppression, with TR = 20.5 msec, TE = 9.0 msec, flip angle = 12^o^, field of view = 44 × 44 mm, imaging matrix = 316 × 243, slice thickness = 0.30 mm, number of excitations = 4, with approximately 42 slices for an acquisition time of 10 min, 31 sec. A pre-contrast acquisition (t = 0 min) was acquired, followed by two sequential post-contrast acquisitions with k-space centered at 5:14 min (t = 5 min) and 15:45 min (t = 15 min) after contrast injection into the dorsal toe of both feet. Reconstructed image spatial resolution was 0.1 mm in plane with 0.15 mm slice thickness.

### Image Analysis

Image analysis was performed using ImageJ software (National Institutes of Health, Bethesda, MD, USA), incorporating custom in-house software developed using Java (Oracle Corp, Redwood Shores, CA, USA), as previously described^33^. Signal intensities were measured from sequential pre- and post-contrast T1-weighted 3D images by manually delineating regions of interest (ROI) over the entire LN in multiple image slices, which were then combined to measure the entire LN volume. ROIs were drawn separately for each pre- and post-contrast time point to account for any bulk motion. The integrated density was then measured as the sum of the pixel values in the ROI for each LN, to take into account both the change in signal intensity and the volume over which it occurs, in the normal size RPNs versus enlarged tumor-draining LPNs^47^. In each case, integrated density was calculated for the whole LN volume, and post-contrast changes in integrated density were measured by subtracting pre-contrast values to reflect LN contrast uptake.

Contrast uptake in the most enhancing or “hot-spot” regions of each LN was also characterized. These pixels could not be isolated using traditional subtraction techniques due to variable misregistration between the pre- and post-contrast images resulting from the manipulation and repositioning required for the manual subcutaneous contrast delivery. Therefore we used a whole LN histogram-based thresholding approach to select only the brightest and presumably most-enhancing pixels in post-contrast images, with signal intensity values higher relative to LN pre-contrast levels. Integrated density was calculated for each time point by summing pixels in the LN meeting a predetermined threshold value.

For comparison of the utility of each contrast agent for popliteal or inguinal LN labeling, the extent of 90^th^ percentile hot-spot labeling at the 5 min timepoint (Figs 3 and 5) was graded. A qualitative scoring system dividing the intensity of contrast uptake into five categories was employed to generate Table 1, ranging from no labeling (−), very weak labeling (+/−), weak labeling (+), moderate labeling (++), to strong labeling (+++).

For visualization of LNs and lymphatic vessels, three-dimensional maximum intensity projections (MIPs) were created using the Philips Extended MR WorkSpace 2.5.3.4 system (Philips Medical Systems, Best, The Netherlands).

### Statistical Analysis

Difference in post-contrast compared to pre-contrast integrated density measures was predicted by side (tumor-draining left and non-tumor-draining right), by post-contrast time point (5 min and 15 min), and by an interaction term to evaluate whether side effects differed by time point. Separate linear mixed effects models for each contrast agent were fitted to control for random mouse effects; pairwise comparisons in the interaction term used the Tukey-Kramer test to control for multiple comparisons. The non-parametric matched pairs Wilcoxon signed rank test was used to compare LN volume data and the proportion of total LN pixels above the 90^th^ percentile in histograms. Analyses were performed separately for the whole LN (based on all LN pixels) and for hot-spot LN regions (based on pixels meeting the 90^th^ percentile threshold. Analyses were performed using SAS v9.4 and JMP v10.0 (SAS Institute, Cary, NC).

## Additional Information

**How to cite this article**: Partridge, S.C. *et al*. Tumor-induced lymph node alterations detected by MRI lymphography using gadolinium nanoparticles. *Sci. Rep*. **5**, 15641; doi: 10.1038/srep15641 (2015).

## Supplementary Material

Supplementary Information

## Figures and Tables

**Table 1:** Detection of contrast agents in lymph nodes.

	Normal Lymph Node			Tumor-draining Lymph Node		
	Gd-DTPA	Gd-LNP	Gd-FVT	Gd-DTPA	Gd-LNP	Gd-FVT
Popliteal lymph node	+	+/−	++	+	+++	++
Inguinal lymph node	+	+	++	−	−	+/−

Symbols. ^+++^strong labeling, ^++^moderate labeling, ^+^weak labeling, ^+/−^very weak labeling, ^−^no labeling.
