# Supplementary material for: Tumor-induced lymph node alterations detected by MRI lymphography using gadolinium nanoparticles
Source: Sci Rep. 2015 Oct 26;5:15641. doi: 10.1038/srep15641 (PMC4620490; doi:10.1038/srep15641)

SUPPLEMENTARY FIGURE LEGENDS

Tumor-induced lymph node alterations detected by MRI lymphography using gadolinium nanoparticles

Partridge, S.C., Kurland, B.F., Liu, C.-L., Ho, R.J.Y., and Ruddell, A.

Supplementary Figure 1. Quantitation of un-thresholded contrast agent uptake into the popliteal lymph nodes.

Integrated density values over each popliteal LN were calculated including all pixels, with no thresholding. Change from pre-contrast integrated density is summarized for groups of 6 mice receiving each contrast agent, with standard error bars displayed at each timepoint. a). The integrated density increases within 5 min after injection of Gd-DTPA in the LPN (#; p=0.02) and RPN (#;p=0.01), as estimated in a linear mixed effects regression model. b). The integrated increases within 5 min after injection of Gd-LNP for the tumor-draining LPN (#; p=0.02), but not for the RPN (p=0.14). There is no significant difference in contrast uptake into the LPN versus RPN for either contrast agent at either post-contrast time point.

Supplementary Figure 2. Determination of optimal threshold to distinguish hot-spots of contrast agent uptake in tumor-draining versus non-draining popliteal lymph nodes.

The optimal threshold to identify differences in contrast agent uptake in hot-spots in the LPN and RPN was identified by calculating the change in integrated density in 5 min

post- minus pre-contrast agent uptake at each thresholding value. The change in integrated density in the RPN was then subtracted from the change in the LPN. The 90th percentile thresholding yields the greatest separation across all three contrast agents, and significantly discriminates Gd-LNP contrast agent uptake between the LPN and RPN (*; p = 0.03; Wilcoxon matched pairs signed rank test).

Supplementary Figure 3. Quantitation of un-thresholded contrast agent uptake into the inguinal lymph nodes.

Integrated density values over each inguinal LN including all pixels, with no thresholding. Change from pre-contrast integrated density is summarized for groups of 6 mice receiving each contrast agent, with standard error bars displayed at each timepoint. a). The integrated density increases within 5 min after injection of Gd-DTPA in the RIN (p=0.007) but not the LIN (p=0.10) as estimated in a linear mixed effects regression model. The contrast uptake is significantly greater in the RIN than the LIN at 15 min post-contrast (*; p = 0.01). b). The integrated density did not significantly increase within 5 min or 15 min after Gd-LNP injection in the RIN or the LIN (p>0.05 for all comparisons). Gd-LNP uptake is significantly greater in the RIN than in the LIN at both 5 min (*; p = 0.004) and at 15 min (*; p = 0.005). c). The integrated density increases within 5 min after injection of Gd-FVT in the RIN (#; p=0.02) but not the LIN (p=0.08). There is no significant difference in contrast uptake between the RIN and LIN at either time point.

Supplementary Fig. 1

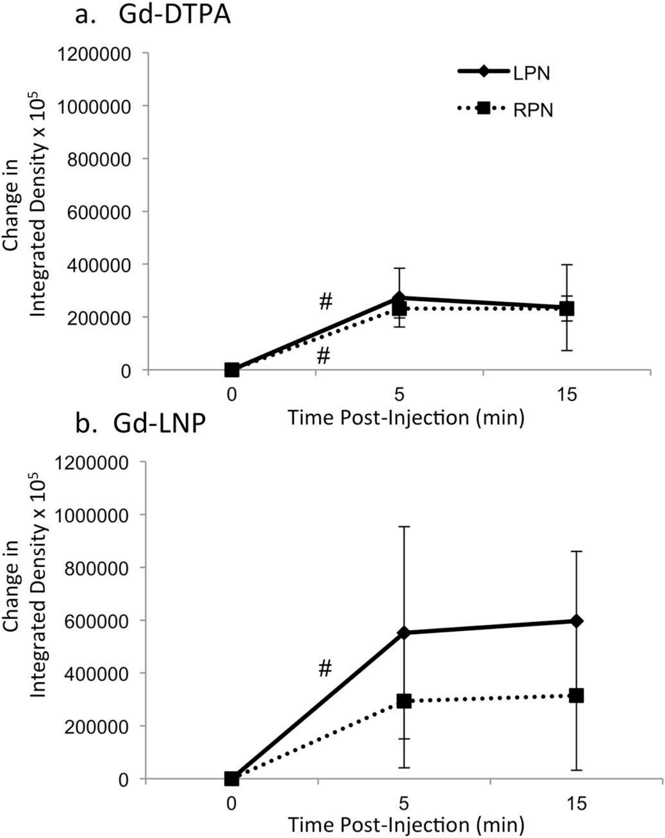

Supplementary Fig. 2

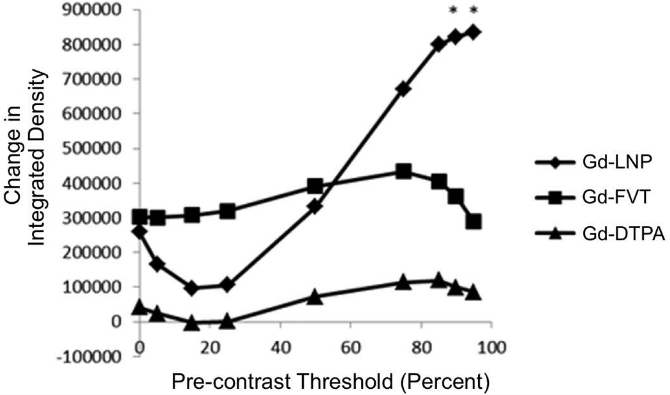

Supplementary Fig. 3

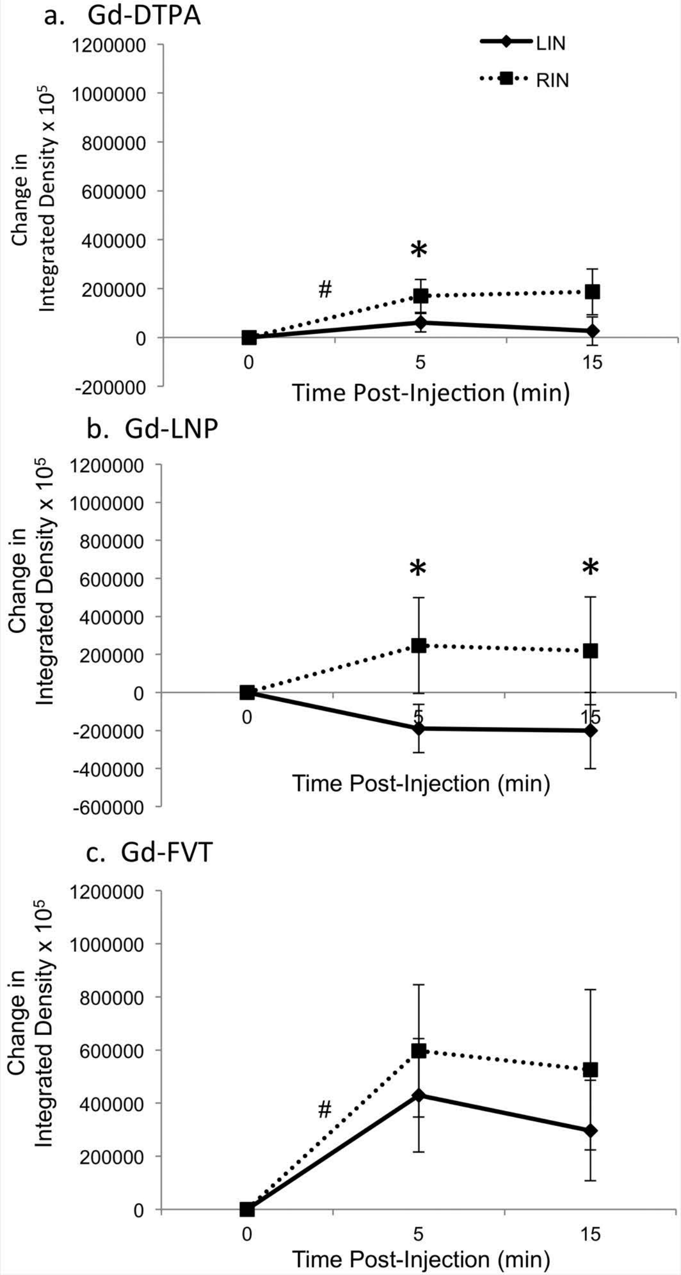

Supplement: Supplementary Information [file srep15641-s1.pdf]
